# Animal-Based Measures for the On-Farm Welfare Assessment of Geese

**DOI:** 10.3390/ani10050890

**Published:** 2020-05-20

**Authors:** Carlo Tremolada, Halina Bielińska, Michela Minero, Valentina Ferrante, Elisabetta Canali, Sara Barbieri

**Affiliations:** 1 Dipartimento di Medicina Veterinaria, Università degli Studi di Milano, 26900 Lodi, Italy; michela.minero@unimi.it (M.M.); elisabetta.canali@unimi.it (E.C.); sara.barbieri@unimi.it (S.B.); 2 Kołuda Wielka Experimental Station, The National Research Institute of Animal Production, 88-160 Janikowo, Poland; kobhg@poczta.onet.pl; 3 Dipartimento di Scienze e Politiche Ambientali, Università degli Studi di Milano, 20133 Milano, Italy; valentina.ferrante@unimi.it

**Keywords:** goose, animal welfare, animal-based measure, inter-observer reliability

## Abstract

**Simple Summary:**

This paper aims to identify animal-based measures of geese welfare for birds raised in commercial meat production systems, and to assess the reliability of these measures. As with other livestock production systems, it is important that geese producers can demonstrate compliance with accepted welfare standards. Presently, there are no welfare measures that have been developed specifically for the geese meat industry. The results showed that plumage dirtiness, twisted wings, and broken/twisted wings are valid and reliable measures of goose welfare and can thus be included in on-farm welfare assessment protocols. Future studies should examine the reliability of other animal-based measures.

**Abstract:**

Currently, no specific animal-based measures (ABMs) protocols are available for geese in commercial meat production systems. Following a critical review of the literature and consultation of experts, seven ABMs, potentially valid and feasible for the on-farm welfare assessment of geese, were identified and then tested in 12 farms in Poland to assess their inter-observer reliability. Two observers conducted the assessment, which was divided into two phases. First, a handling test assessed the human–animal relationship (HAR), and a 100% inter-observer reliability was achieved by the observers when evaluating the attitudes of stockpeople and the reactions of geese to humans. Next, an animal inspection was conducted, and the observers simultaneously and independently visually evaluated 100 randomly selected geese per farm and assessed whether the selected ABMs could be identified. In terms of inter-observer reliability, high correlation coefficients were found for plumage dirtiness (ρ = 0.745; *p* < 0.01), twisted wings (ρ = 0.890; *p* < 0.001), and broken/twisted wings (ρ = 0.858; *p* < 0.001). The results showed that plumage dirtiness, twisted wings, and broken/twisted wings are valid and reliable measures. Further research should address the reliability of ABMs of geese in other types of production systems.

## 1. Introduction

Social awareness of farm animal rearing conditions has increased in recent years [1], and animal welfare has become a prerequisite for companies that aim to develop high-quality and sound animal products for the global market [2].

Welfare is a multidimensional concept that includes the consideration of both the physical and mental states of animals [3], so its assessment should be based on measures developed using species-specific parameters.

Two broad categories of measures can be used to assess animal welfare at the on-farm level: Resource-based and animal-based (ABMs) [3]. Rearing conditions have previously been evaluated using assessment methods mainly focused on resources (e.g., feeding systems, space availability, and housing) and on management factors [4]. ABMs relate directly to the animal rather than to its living environment [3], and can thus be obtained from various housing conditions and uniquely used to compare farming systems, enabling the standardization of evaluation methods. Therefore, valid, reliable, and feasible measures should be identified so on-farm welfare assessment protocols can be developed [5].

Integrating ABMs into on-farm welfare assessment protocols also supports the European Commission’s call for a more outcome-based approach to integrating the EU’s animal welfare regulatory framework [3]. The protocols should also be science-based to ensure they comply with legislation or standards that guarantee the welfare of farm animals.

Few protocols for assessing animal welfare at the farm level that include valid, reliable, and feasible ABMs are currently in place. Welfare assessment protocols for poultry have been developed [6,7], but no ABM-based protocols assess the welfare of geese under commercial production.

Geese were first domesticated over 3000 years ago and are now mainly reared for meat and down production [8]. Geese can be raised successfully on pasture with little equipment and simple housing facilities in areas where green grass is available for a large part of the year. Under free-range conditions, adult geese only require indoor housing or shelter in winter or during stormy weather [9]. Geese are excellent grazers, well adapted to most environments, and forage extensively, and thus are particularly easy to rear compared to other poultry species, while maintaining a high and efficient growth rate [10].

Despite these positive aspects, goose production is marginal in many countries, and is only economically relevant in Asia and Central Europe [11,12].

There are some regulations on the welfare of the goose mainly about the production of feathers or the production of foie gras [13,14]. Research into the effects of management practices on the welfare of geese raised for meat production is more limited than other poultry species, which prevents specific animal-based measures from being identified and subsequently welfare assessment protocols from being developed.

The approach and the methodology for identifying a set of specific animal-based measures to assess the welfare of fattening geese under commercial conditions are described in this study, and the most pressing welfare issues are identified. The resulting list of measures was tested on-farm to assess inter-observer reliability.

## 2. Materials and Methods

The welfare measures were determined through two stages: A set of ABMs were first selected based on their validity and feasibility for on-farm use, and inter-observer reliability was then tested after the observers were trained to use the ABMs.

### 2.1. Selection of Animal-Based Measures

In the first stage of the project, a critical review of the literature was conducted to identify the key studies addressing animal-based welfare measures in birds raised in commercial meat production systems. Various databases (Web of Science, CAB Abstracts, and PubMed) were searched using keywords such as “feather pecking”, “gait”, “beak trimming”, “mutilations”, “feathers conditions”, “feathers harvesting”, “twisted wings“, “angel wings”, “broken wings”, human-animal relationship”, “behavior test”, “animal-based”, “animal welfare”, “indicator”, “measure”, “on-farm”, “inter-observer reliability” combined with “goose”, “geese”, “duck”, “poultry”.

The search resulted in about 60 citations from which studies addressing geese production and animal-based welfare measures were initially selected. Due to the limited number of studies on geese, relevant publications on welfare measures developed for ducks and poultry (turkeys and broilers) were included. We excluded any studies that solely concentrated on resource-based or management-based measures. After the selection process, a total of 22 papers and two welfare assessment protocols (Welfare Quality^®^ and AWIN for turkeys), published between 1973 and 2019, satisfied the search criteria, identifying 12 ABMs.

In a second phase, a one-day consultation with scientists from the National Research Institute of Animal Production of Poland, internationally acknowledged for their expertise in goose farming was carried out. In order to define the most promising ABMs to be tested on-farm, scientists were asked to evaluate each ABM for its validity, in terms of the extent to which its scores represent the variable they are intended to measure [15], and feasibility, i.e., practicality for on-farm assessment, particularly when applied to semi-extensive rearing conditions. After group discussion, a synthesis was made, where scientists reached a consensus on validity and feasibility of seven ABMs. The final selected ABMs were as follows.

Plumage dirtiness describes a condition in which the feathers covering the breast area of geese are soiled with faeces or dirty litter. Dirty feathers can lose their protective properties, which has a significant effect on bird welfare [3,16]. The plumage conditions of commercially reared birds are routinely assessed through the Welfare Quality^®^ assessment protocol for broilers and in the AWIN for turkeys [6,7].

Twisted wing is a musculoskeletal disorder in which the flight feathers of one or both wings of a bird twist away from the body, mainly during growth [17,18]. As reported in [19], welfare problems such as a high stocking density, a fast growth rate, and genetic selection are the main factors contributing to the twisted wing in white geese. Visually examining the wing posture can provide a feasible assessment measure of the presence of wing deformities in commercially reared geese [19].

Feather pecking is an abnormal behavior involving rapid and vigorous pecking directed towards the feathers of another bird, resulting in a poor plumage condition, patches of feather loss, and skin injuries [20]. Some researches into waterfowl [20,21,22], and other poultry have investigated the feasibility of measuring feather pecking and its validity in commercial farms [6,7,23].

Broken wings are a valid and feasible animal-based measure that can be used to assess the welfare of geese submitted to inappropriate feather collection and poor catching or handling [14,24].

Immobility can result from health problems, severe walking deficiency and abnormalities in standing posture, and gait in waterfowl [13,20,25]. Measures such as posture and walking ability are commonly used in assessments of duck and geese welfare [20,26,27]. A lack of mobility is relevant to poultry welfare and the ability to walk is routinely assessed as part of the Welfare Quality^®^ assessment protocol for broilers and in the AWIN for turkeys [6,7].

Feather irregularities, bloody feather quills, and skin injuries in the ventral-breast area are indicators that feathers were collected inappropriately. Consequently, they can be used to assess the forcible removal of unripe feathers during commercial collection [14].

Handling tests are designed to measure animals’ reactions to human beings, and the attitudes and behavior of stockpeople during routine interactions with animals [28]. These interactions are associated with animal welfare parameters such as levels of fear, stress responses, and productivity [29]. Methodologies for assessing how comfortable animals are with people have been developed and applied, and human–animal relation tests are the tools most commonly included in welfare assessment protocols (e.g., avoidance distance test in the Welfare Quality^®^ assessment protocol for poultry [6]).

### 2.2. Birds and Management

The refined set of seven ABMs was tested in 12 semi-extensive farms located in the same geographical region in Poland, which had an average flock size of 6450 geese per farm. The study was conducted in the summer with steadily good weather conditions. Geese were housed in one single flock in each farm. The animals were White Koluda fattening geese ranging from 10 to 12 weeks of age at the time of the evaluation and the age of slaughter was at 15 weeks. The stocking density was between 0.42 and 1.32 birds/m^2^. Each farm had one or two indoor houses, which included using chopped straw as bedding, automatic drinkers, manual feeders, artificial light, and windows. A fenced pasture was available for the geese to graze on at each farm during daylight hours while they were kept indoors at night. Their diet consisted of grasses with supplemental feeding of grains and concentrated feeds twice a day. All the farms participating in the study were part of a controlled supply chain subject to routine audits.

### 2.3. Observers

The ABMs were evaluated by one of the authors (Obs-1) and a veterinarian (Obs-2), both of them experienced in goose behavior and welfare, although Obs-2 had limited experience with ABMs. Before carrying out the on-farm evaluation, Obs-2 underwent a training period to learn how to perform and score all the selected measures. The training was carried out by a team of experts, which included the Obs-1 and consisted of two phases: First, e-learning and then discussion in a virtual meeting room. The e-learning phase was developed in order to reduce time and costs related to the training, without losing accuracy in the assessment. Each welfare measure was transferred into a learning object organized in different categories: Description, how to assess, how to score, and some practical examples. Then, a virtual meeting room, enabling doubts to be clarified, was performed in order to acquire a uniform level of skills necessary to perform the assessment accurately and reliably.

The training ended with an assessment to evaluate the learning and its effectiveness, and consisted of 20 questions (including videos and/or pictures); the training was considered complete when the assessor achieved over 70% of correct answers.

### 2.4. Data Collection

During the handling test, one observer asked the stockperson to walk inside the pen and to lead a group of geese (ranging from a minimum of 20 to a maximum of 30 birds) for 1 min from a defined place (point A) of the fenced pasture to another one (point B), using his/her routine handling techniques. During the test, only the stockperson was close to the animals while the observers remained at a distance (approx. 15 m), to ensure the test was properly conducted.

The remaining six ABMs were assessed by an animal inspection after the handling test, to avoid excessive disturbance of the animals. The inspection was carried out in the indoor houses and in the pasture, depending on where geese were at the time of inspection. Before starting the inspection, the general status of the animals was observed and both observers waited until the flock settled down.

The pasture and the indoor house (if available for geese during daylight hours) were divided along predetermined paths, covering the full area the geese were located in at the time of inspection. Obs-1 and Obs-2 walked slowly alongside the paths in a random order, stopping every 12 steps. Both observers simultaneously and independently visually evaluated a maximum of four randomly selected geese every time they stopped, maintaining a distance of about 1 m from the inspected animals. At the end of the assessment, a total of 100 geese were assessed in each farm. Geese were not restrained nor individually handled by the stockperson or by the observers. Data were recorded using specific recording sheets by both observers. The assessment of each measure and of the scoring system is described in Table 1.

### 2.5. Statistical Analysis

The mean numbers of geese within each animal-based measure category were calculated for each observer. Prevalence indices for all of the ABMs were calculated. The prevalence index is the absolute difference between the agreed numbers for the two categories, divided by the total number of animals (prevalence index = │a-d│/*n*), where a is the number of agreed-upon animals in one of the categories and d is the number for the other categories, and *n* is the total number of possible agreements, i.e., the number of animals. A prevalence index of 0 indicates a completely balanced population, while an index of 1 indicates a homogenous population in which only one of the categories is represented [30].

A Kolmogorov–Smirnov test was applied to the continuous variables to assess the normality of data distribution. Though Cohen’s kappa (κ) is the elective measure of an inter-rater agreement for categorical scales when there are two raters, it is not considered reliable in case of small or skewed populations similar to that used in this study. Therefore, the inter-observer reliability was analyzed by calculating Spearman’s correlation ranks.

## 3. Results

The data were not normally distributed; therefore, nonparametric tests were used to analyze the data. The results from the handling test showed that both Obs-1 and Obs-2 reached 100% agreement in evaluating the stockperson’s attitude when dealing with the animals, and the reactions of geese to humans, in all the assessed farms. The mean prevalence values of the selected ABMs are given in Table 2.

Only the ABMs with a mean value of prevalence >0 were analyzed to calculate the inter-observer reliability. These were plumage dirtiness, twisted wings, and broken wings. The mean values were consistent between observers for the considered ABMs with the exception of broken wings (Obs-1 = 0; Obs-2 = 0.5). The prevalence of broken wings was very low for both observers, and a new measure, “broken/twisted wings,” was created by merging broken and twisted wing observations.

The inter-observer reliability showed high correlation coefficients for plumage dirtiness (ρ = 0.745; *p* < 0.01; prevalence index = 0.36), twisted wings (ρ = 0.890; *p* < 0.001; prevalence index = 0.95), and broken/twisted wings (ρ = 0.858; *p* < 0.001; prevalence index = 0.95), with a balanced prevalence for plumage dirtiness.

## 4. Discussion

In this study, we investigate the use of species-specific animal-based measures for the assessment of goose welfare in commercial production systems. Currently, no other available research evaluates the validity and the reliability of ABMs on commercially raised geese, while specific research on other poultry species can be found in the scientific literature [6,7,31,32].

In the initial step, a list of relevant ABMs were selected from the literature and from available science-based welfare assessment protocols for other meat poultry (e.g., Welfare Quality^®^ assessment protocol for poultry [6]). Some ABMs (broken bones and feather irregularities) were included to account for expected differences between geese and other poultry species, based on specific management procedures affecting animal welfare (e.g., live-plucking [14]). As geese are commonly kept in social groups in extensive or semi-extensive rearing conditions, and as they are large and have a flighty nature, all the ABMs in the study were adapted to be applied with no or minimal handling of the birds.

The assessment of the human–animal relationship using specific behavioral tests is common in protocols intended to evaluate animal welfare at the farm level [6,7]. As reported [28], the nature of the interactions between stockpeople and farm animals is an important component affecting the animals’ welfare: Positive interactions can reduce stress and fear in animals and increase productivity, while aversive human–animal relationships may reduce their performance [33]. However, no specific HAR tests have been validated for geese. In this study, we aimed to evaluate the HAR in commercially raised geese using a handling test. A central characteristic for any measurement tool is consistency in measurements when applied by different assessors [34]. According to our results, both Obs-1 and Obs-2 reached a 100% inter-observer reliability in evaluating the human–animal relationship during the handling test. In terms of feasibility, the test appeared to be practical when applied to farms, where the stockperson moved a group of geese from one point to another in the home pen as they would in daily management procedures.

The results for the ABMs assessed during the animal inspection were consistent across Obs-1 and Obs-2 for plumage dirtiness, twisted wings, and broken/twisted wings (Table 2).

This study, however, is subject to some limitations. First, differences were found across observers for the prevalence of broken wings, and in a few cases for twisted and broken wings. Obs-2 had less experience in welfare assessment using ABMs, and scored the two wing-related measures (twisted wings and broken wings) similarly, thus reporting a low level of agreement with Obs-1 (an expert in the use of animal-based measures). The interpretation of twisted wings may have been unclear, as they were ascribed as broken by the less experienced assessor (Obs-2). Then, the inter-observer reliability of broken wings was thus proven to be difficult to achieve, as extensive training was required to address the lack of experience of Obs-2. Although the assessment was carried out by observers with different experiences in assessing geese welfare by using ABMs, our findings confirm that on-farm training is essential for achieving good reliability [35].

Our findings suggest that the high prevalence of plumage dirtiness can be related to poor litter conditions, as supported by Saraiva [36], and hence provides information regarding goose welfare at the farm level. The high SE values also suggest high variability among farms reflecting different management conditions, particularly concerning housing design and litter management. The plumage condition is important for thermoregulation, and when the feathers are wet or soiled by litter they may lose their protective properties, thus having negative effects on the birds’ welfare [6,37]. In addition, when the down is soiled by faeces or dirty litter, there may be negative consequences on its color, which is an important factor affecting the down value.

Few studies have reported the prevalence of twisted wings in commercially raised geese [19]. According to our study, a small number of geese showed wing deformity. Our findings are supported by Lyn, who reported that slight twisted wings returned to a normal position during 10 to 14 weeks of age when secondary feathers reached full maturity [19]. Moreover, our study was conducted in extensive or semi-extensive rearing conditions in which geese have access to pasture, which provides them with all the required nutrients, and thus may not suffer from vitamin deficiency and elevated protein concentration in the diet, which are suggested causes leading to this deformity [18].

The prevalence of feather irregularities, feather pecking, and immobility was low because these ABMs have never been found on the assessed farms. As pointed out by Burn [30], “the prevalence of certain observations reduces the reliability ratings”. Therefore, when evaluating inter-observer reliability, scientists should consider the prevalence of the different ABMs in the population assessed. An unbalanced prevalence could lower the reliability statistics, and reporting the prevalence index alongside other measures of agreement showed that for some ABMs the populations were too homogenous for conclusive reliability ratings. Although feather irregularities, feather pecking, and immobility have been identified as important measures for defining poultry welfare at the farm level [6,7,14], the reliability for these ABMs was difficult to prove. However, our results suggest an agreement between Obs-1 and Obs-2, as neither of them found any of these measures in any assessed farms.

In the present study, the selected ABMs were identified based on key studies of animal-based measures in commercial waterfowl and poultry production.

The lower prevalence of the selected ABMs compared to those in other poultry species may indicate the better welfare conditions of geese, probably due to different housing and management practices. All the assessed farms were free-range, and the geese could, therefore, express their natural behavior (e.g., grazing and movement activity), and more space was available than in intensive farming systems. High stocking density and poor environmental conditions are significant factors affecting the welfare of poultry [32].

Despite the low frequencies of some measurements, the preliminary results of our study suggest that the identified animal-based measures may offer a valid, reliable, and feasible tool for assessing the welfare of geese in extensive or semi-extensive rearing conditions.

Future testing would require the selection of a more diverse geese population. This can be achieved by including breeding farms, which nowadays tend towards intensive, closed conditions as well as farms in different countries and geographical areas (e.g., China) where different husbandry systems are applied.

## 5. Conclusions

As the initial step in the development of a specific welfare assessment protocol, this study is aimed at identifying potentially valid, reliable, and feasible animal-based indicators applicable to the on-farm welfare assessment of geese.

The handling test proved to be practical on farms, with the stockperson moving a group of geese from a point to another in the home pen, as they would in daily management procedures. Further studies are needed to assess the possible variation in the agreement between the observers and to evaluate if the test is sensitive to fluctuations in the welfare status of geese among farms.

The inter-observer reliability was confirmed for three ABMs. Our results show that plumage dirtiness, twisted wings, and broken/twisted may be valid and reliable measures of goose welfare. Further studies can consider variations in reliability and verify if the selected ABMs are sensitive enough for identifying and quantifying welfare differences.

The low prevalence of feather irregularities, feather pecking, and immobility meant we could not confirm their reliability.

The selected ABMs can be developed into a valid and feasible tool for the on-farm evaluation of geese welfare, although adequate training is required to produce reliable data when used by different observers, and further studies are needed to confirm reliability. In addition, the size of the sample should be increased so the reliability ratings can be fully implemented, and to determine whether possible differences in frequency are due to the effect of the sample size or to the housing conditions.

## Figures and Tables

**Table 1:** Description of each animal-based measure (ABM) and of the scoring system.

ABMs	Description	Two-Point Scale Scoring System	Level
Handling Test	The observer evaluates the stockperson’s attitude when dealing with the animals and the reactions of geese to humans.	Stockperson’s attitude: 0 = approaching and speaking in a gentle voice, walking slowly, touching the animals gently with a stick. 1 = shouting loudly, handling or hitting the animals forcefully with a stick. Animals’ reaction: 0 = >50% of geese showing normal behavior. 1 = >50% of geese showing adverse or fear reactions, avoidance reactions, loud vocalizations, or wing flapping.	G ^1^
Plumage dirtiness	The observer visually inspects the ventral-breast area. Presence of mud attributed to current weather conditions is not considered as plumage dirtiness.	0 = no signs of dirt or slight dirt of plumage covering less than 50% of the ventral-breast area. 1 = more than 50% of the plumage of ventral-breast area is contaminated with dirt or feces ^3^.	I ^2^
Feather irregularities	The observer visually inspects the ventral-breast area.	0 = smooth feathers with no signs of disturbance. There is no evidence of skin lesions to any part of the ventral-breast area. 1 = presence of bloody feather quills, skin injuries, or feathers that are different in maturity compared to those on the rest of the body that indicate the forcible removal of unripe feathers.	I
Broken wings	The observer visually inspects the wings posture.	0 = both wings are in a normal posture. 1 = one or both wings are dangling.	I
Twisted wings	The observer visually inspects wings posture.	0 = both wings are in a normal posture. 1 = one or both wings are twisted with primary feathers projecting away from the body (angle of deviation ranging from <30 to >60°).	I
Feather pecking	The observer visually inspects the plumage condition of neck and back regions.	0 = neck and back regions are covered with smooth and feathers that fit neatly along the body. 1 = feathers are disturbed, broken, or even torn out. Presence of patches of lost feathers (diameter >5 cm) or skin damages.	I
Immobility	The observer visually evaluates the birds’ ability to walk.	0 = normal walking ability. 1 = complete immobility, severe abnormality in walking, or reluctance to move.	I

^1^ The ABM is scored at group level; ^2^ the ABM is scored at individual level; ^3^ presence of mud was not considered as plumage dirtiness.

**Table 2:** Mean (± SE) prevalence of geese (number of observations, *n* = 100) within each ABMs category assessed during animal inspection for Obs-1 and Obs-2 in all the inspected farms (*n* = 12).

ABMs	Observer 1 Mean (SE)	Observer 2 Mean (SE)
Plumage dirtiness	31.66 (25.46)	44.25 (33.17)
Twisted wings	2.25 (2.05)	2.41 (1.78)
Broken wings	0 (0)	0.51 (0.26)
Broken/twisted wings	2.25 (2.05)	2.91 (2.23)
Feather irregularities	0 (0)	0 (0)
Feather pecking	0 (0)	0 (0)
Immobility	0 (0)	0 (0)
